# Curcumin‐Loaded GelMA Microspheres Alleviate Osteoarthritis: Transcriptomic Evidence for Immune Microenvironment Remodeling and ECM Homeostasis Restoration

**DOI:** 10.1111/cbdd.70373

**Published:** 2026-08-02

**Authors:** Bin Jiang, Xin Jiang, Shaobo Li, Hao Niu, Wenbin Ji

**Affiliations:** ^1^ Rehabilitation Department Qilu Hospital of Shandong University (Qingdao) Qingdao Shandong China; ^2^ Sports Medicine Department Qilu Hospital of Shandong University (Qingdao) Qingdao Shandong China

**Keywords:** cartilage regeneration, curcumin, immunomodulation, microspheres, osteoarthritis, transcriptomics

## Abstract

Osteoarthritis (OA) progression is driven by inflammatory mediators and immune dysregulation within the joint. Curcumin (Cur) possesses multi‐target therapeutic potential. However, its clinical application is limited by poor solubility and a short half‐life. In this study, we developed injectable Cur‐loaded GelMA microspheres (Cur‐MS) and evaluated their effects in IL‐1β stimulated chondrocytes, cartilage organoids, and a monosodium iodoacetate (MIA) induced rat OA model, complemented by public transcriptomic analysis (GSE114007). The Cur‐MS demonstrated uniform size distribution, favorable biocompatibility, and sustained curcumin release. Treatment with Cur‐MS significantly reduced chondrocyte apoptosis, reactive oxygen species levels, and hypertrophic markers; restored COL‐II and ACAN synthesis; and downregulated MMP13 and inflammatory gene expression. In vivo, Cur‐MS improved joint space, alleviated pain, decreased synovial CD68 positive macrophage infiltration and levels of IL‐6 and TNF‐α, and enhanced chondrogenic gene expression. Public transcriptomic data corroborated these findings, revealing upregulation of MMP13 and downregulation of SOX9 in OA cartilage, consistent with our experimental targets. Collectively, this study provides the first multi‐model evidence combined with transcriptomic validation, demonstrating that Cur‐MS not only directly protects chondrocytes and restores extracellular matrix homeostasis but also modulates the joint inflammatory immune microenvironment. These findings suggest that Cur‐MS represents a promising locally sustained‐release therapeutic strategy for OA.

## Graphical abstract

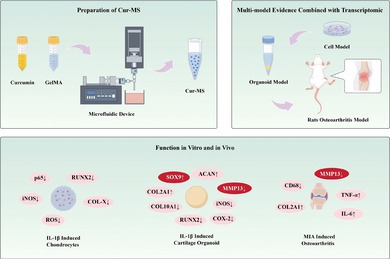

## 1 Introduction

Osteoarthritis (OA) is a prevalent degenerative joint disorder characterized by progressive cartilage degradation, subchondral bone sclerosis, and synovial inflammation, which collectively result in chronic pain and functional impairment (Hunter and Bierma‐Zeinstra 2019; Bennell et al. 2021; Han et al. 2024; Duong et al. 2023). Currently, clinical management of OA primarily focuses on alleviating pain and enhancing joint function. Common therapeutic approaches include exercise therapy, oral administration of nonsteroidal anti‐inflammatory drugs, and local injections of analgesic and lubricating agents (Safran‐Norton et al. 2019; Pelletier 1999; Yuan et al. 2023; Weng et al. 2023). However, these interventions are limited to providing symptomatic treatment and do not modify disease progression or facilitate cartilage repair. For localized cartilage lesions or advanced stages of the disease, surgical options such as arthroscopic debridement, bone marrow stimulation, and autologous chondrocyte implantation are employed (White et al. 2021; Colombini et al. 2023). Nonetheless, these techniques often result in the formation of fibrocartilage, which possesses inferior mechanical properties, and are associated with limitations including donor site morbidity, chondrocyte dedifferentiation, and suboptimal integration of repair tissue (Hwang and Elisseeff 2009). In cases of severe OA, joint replacement surgery remains the definitive treatment. Although effective, it entails considerable surgical trauma, high costs, and limited implant longevity (Longo et al. 2024; Jain and Parashar 2022). Therefore, there is an urgent need to deepen the understanding of the pathological mechanisms of OA and to explore new treatment strategies that can effectively delay or even reverse cartilage degeneration.

The pathological progression of OA is driven by a complex, self‐perpetuating inflammatory microenvironment within the joint (Hu et al. 2021; Li et al. 2022). Pro‐inflammatory cytokines, particularly interleukin‐1 beta (IL‐1β), play a pivotal role in this process by disrupting the homeostatic balance of joint chondrocytes (Kapoor et al. 2011; Yang et al. 2018). Previous studies have demonstrated that IL‐1β markedly upregulates the expression of catabolic enzymes, including matrix metalloproteinases (MMPs), which directly degrade critical extracellular matrix (ECM) components such as type II collagen (COL‐II) and proteoglycans (Keller et al. 2023; Xu et al. 2023; Zhang, Yan, et al. 2024; Hu and Ecker 2021). Concurrently, IL‐1β suppresses the synthesis of these essential matrix proteins, thereby accelerating cartilage degradation (Wang et al. 2023; Tu et al. 2019; Chien et al. 2020). Furthermore, within the immune microenvironment of the joint, infiltrating macrophages and T cells secrete substantial amounts of inflammatory mediators including IL‐1β and IL‐6 (Zhang et al. 2025; He et al. 2026). These mediators disrupt the homeostatic balance between synthesis and degradation of ECM, resulting in excessive degradation of COL‐II and ACAN, thereby accelerating cartilage destruction. Consequently, pharmacological treatment strategies for OA have identified the inhibition of these inflammatory mediators' biological activity and the remodeling of the joint immune microenvironment as central targets for cartilage protection.

In recent decades, cartilage tissue engineering approaches utilizing chondrocytes have emerged as a promising alternative for the repair of damaged cartilage (Nordberg et al. 2024; Bhattacharjee et al. 2015). A widely accepted and effective in vitro model for cartilage differentiation is the three‐dimensional pellet or micromass culture system (Lee et al. 2021; Yoon et al. 2020). This method facilitates cell interactions during cultivation, promoting the formation of a rich ECM (Zheng et al. 2022). Nevertheless, a significant challenge in translating these engineered constructs into clinically effective therapies is the hostile inflammatory microenvironment present in OA joints. The implanted tissue‐engineered cartilage is highly vulnerable to inflammatory mediators that contribute to the degradation of native cartilage (De Roover et al. 2023; Ruiz‐Fernández et al. 2022). Exposure to cytokines such as IL‐1β not only inhibits matrix synthesis but may also induce undesirable hypertrophic differentiation and apoptosis, ultimately resulting in the failure of the regenerated tissue (Cheung et al. 2003). Consequently, the development of effective strategies to protect tissue‐engineered cartilage from inflammatory insults is essential for the successful regeneration of cartilage.

Curcumin (Cur), a natural polyphenol derived from turmeric, has attracted considerable attention owing to its potent anti‐inflammatory, antioxidant, and cartilage‐protective effects (Sadeghi et al. 2023; Zia et al. 2021; Abd El‐Hack et al. 2021). These multifaceted actions are mediated through the modulation of multiple signaling pathways. Notably, Cur functions as an inhibitor of the nuclear factor kappa‐B (NF‐κB) pathway, a central regulator of inflammation (Zhang, Weng, et al. 2024; Zamanian et al. 2024). It suppresses the expression of various inflammatory and catabolic genes by preventing the degradation of IκBα and the subsequent nuclear translocation of the p65 subunit (Chen et al. 2019; Lan et al. 2023). Despite these advantages, Cur encounters considerable obstacles in clinical application owing to its intrinsic characteristics. Specifically, Cur exhibits very low aqueous solubility, undergoes rapid metabolic degradation in vivo, and demonstrates poor bioavailability. These factors hinder the maintenance of its stability and therapeutic concentration within the body. Consequently, there is a critical need to develop more efficient drug delivery systems capable of achieving localized, sustained, and targeted release of Cur within the joint cavity.

In recent years, various intra‐articular delivery strategies for curcumin have been investigated to address its poor solubility and rapid in vivo clearance. Although nanoemulsions enhance curcumin dispersibility and cellular uptake, they do not fundamentally resolve the rapid clearance of small‐molecule drugs from the joint cavity, resulting in a limited duration of therapeutic concentration following a single injection (Hridayanka et al. 2025). Thermosensitive chitosan hydrogels loaded with polycaprolactone microspheres can achieve gel‐state retention within the joint cavity (Kalantarnia et al. 2025). However, their complex multi‐component composition complicates formulation development and poses challenges for clinical translation. The PEG‐GelMA composite microgel system has demonstrated dual in vitro effects by promoting mesenchymal stem cell chondrogenic differentiation and inhibiting chondrocyte inflammatory responses (Sun et al. 2022). Nevertheless, the incorporation of PEG increases system complexity, and the study lacks a comprehensive evidence chain extending from cellular mechanisms to three‐dimensional tissue validation and animal models. Considering these factors, the present study employs methacrylated gelatin (GelMA) as the sole microsphere carrier for curcumin. GelMA integrates the bioactivity of natural gelatin with the tunability of synthetic materials. Additionally, GelMA microspheres can be produced at scale with uniform particle size and batch‐to‐batch consistency via microfluidic technology. GelMA also exhibits favorable biocompatibility and biodegradability, minimizing the risk of long‐term foreign body reactions. Finally, compared to composite systems, the simpler single GelMA matrix structure facilitates formulation quality control and enhances prospects for clinical translation.

Based on the above advantages, we propose a novel therapeutic approach involving the intra‐articular delivery of Cur‐loaded GelMA hydrogel microspheres (Cur‐MS) to achieve both potent anti‐inflammatory and cartilage‐protective effects. Initially, Cur‐MS were fabricated with optimized drug loading through systematic concentration screening and comprehensive material characterization. Subsequently, a rigorously designed stepwise experimental framework was employed. In vitro investigations utilizing both chondrocyte cultures and cartilage pellet models thoroughly assessed the inhibitory effects of Cur‐MS on IL‐1β‐induced chondrocyte apoptosis, oxidative stress, activation of inflammatory signaling pathways, and matrix catabolism. Concurrently, their efficacy in promoting cartilage matrix synthesis and preserving chondrocyte phenotype was validated. The therapeutic potential of Cur‐MS was confirmed in vivo using a monosodium iodoacetate (MIA) induced rat knee OA model via local intra‐articular injection. Finally, we analyzed the publicly available RNA‐seq dataset GSE114007, which comprises human knee articular cartilage samples comparing OA to normal conditions, and identified genes exhibiting significantly altered expression in this study. The results demonstrate that Cur‐MS significantly ameliorate OA joint cartilage degeneration, effectively inhibit cartilage matrix degradation, and attenuate the inflammatory microenvironment. These findings substantiate the potential of Cur‐MS as an efficient, targeted, and safe drug delivery system, highlighting their broad clinical applicability in the comprehensive management of OA.

## 2 Materials and Methods

### 2.1 Preparation of Cur‐MS

Cur was initially dissolved in dimethyl sulfoxide (DMSO) to prepare a 10 mM stock solution, which was aliquoted and stored at −20°C in the dark. Initially, prepare a 10% (w/v) GelMA solution containing 0.25% (w/v) lithium phenyl‐2,4,6‐trimethylbenzoylphosphinate (LAP) photoinitiator, and sterilize it by filtration through a 0.22 μm membrane. Subsequently, incorporate Cur solutions into this mixture to achieve final concentrations of 0, 100, 300, and 500 μM as the water phase for microsphere preparation. Activate the microfluidic device and perform the experiment using a microfluidic chip. Begin by initiating the oil phase injection pump, setting the flow rate to 20 μL/min. Upon establishing a stable and continuous flow of the oil phase (fluorinated oil, Luxue‐FO‐30) within the channel, commence the water phase injection pump loaded with the Cur and GelMA mixture, adjusting the flow rate to 5 μL/min. The shear forces generated between the water and oil phases facilitate the stable formation of monodisperse water phase droplets at the chip's collection outlet, each uniformly encapsulating cells. The droplets collected in the collection tube should be immediately placed under a 405 nm LED ultraviolet lamp for curing, with a light intensity of 20 mW/cm^2^, and irradiated for 1 min. This irradiation triggers LAP photoinitiated free radical polymerization, crosslinking the GelMA to form structurally stable Cur‐MS. Subsequently, add a demulsifier (Luxue‐MDF‐10) to the collection tube at a 1:1 volume ratio, gently invert to mix, and allow the mixture to stand for 5 min to enable complete release of the microspheres from the oil phase into the lower aqueous phase. Carefully aspirate the upper oil phase and demulsifier mixture, retaining the lower aqueous phase containing the microspheres. Add an appropriate volume of physiological saline to the collected solution, gently resuspend, and oscillate at low speed for 1 min. Centrifuge the suspension at 800 rpm for 3 min and discard the supernatant. Repeat the washing procedure three times to thoroughly remove residual oil phase, demulsifier, and un‐crosslinked GelMA monomers. Ultimately, obtain the purified Cur‐MS.

### 2.2 Material Characterization of Cur‐MS

#### 2.2.1 Appearance and Particle Size

Freshly prepared microspheres were collected, washed with phosphate‐buffered saline (PBS), and subsequently freeze‐dried. Following gold sputter coating, the surface morphology of the microspheres was examined using a scanning electron microscope (SEM; ZEISS Sigma 360, Germany) operated at an accelerating voltage of 3 kV. In a separate procedure, fresh wet microspheres were placed in a culture dish and imaged using an optical microscope. The diameters of the microspheres were measured with ImageJ software to determine the average particle size and particle size distribution.

#### 2.2.2 Swelling Performance

Freeze‐dried Cur‐MS with varying concentrations was weighed and recorded as W_d. Subsequently, the microspheres were immersed in PBS at pH 7.4 and 37°C, with samples removed at 24‐h intervals. After gently blotting surface moisture using filter paper, the microspheres were weighed, and the weight was recorded as W_w. Once the weight stabilized and no longer exhibited changes, the final weight was utilized to calculate the swelling ratio.

Swelling Ratio=W_w−W_dW_d×100%

#### 2.2.3 In Vitro Degradation

Accurately weigh varying concentrations of lyophilized Cur‐MS, recording the initial weight as W_0. Subsequently, immerse the microspheres in 1% type I collagenase prepared in PBS and incubate at 37°C on a constant temperature shaker set to 100 rpm. At predetermined time points of 1, 3, 7, 14, 21, and 28 days, retrieve the microspheres. Following lyophilization, reweigh the samples and record the mass as W_t. The degradation rate of the microspheres is then calculated based on the initial mass W_0.

Degradation rate%=W_tW_0×100%

#### 2.2.4 In Vitro Release Rate Detection of Cur

Ten milligrams of microspheres at varying concentrations were placed into dialysis bags with a molecular weight cutoff of 14 kDa. Each bag was filled with 2 mL of PBS at pH 7.4 containing 0.1% Tween‐80, serving as the release medium. After sealing, the dialysis bags were transferred into 15 mL centrifuge tubes and incubated in a constant temperature shaker set at 37°C and 100 rpm. At 1, 3, 7, 14, 21, and 28 days, the entire release medium was collected and replaced with an equal volume of fresh medium. The Cur concentration in the collected release medium was quantified using high‐performance liquid chromatography, and the cumulative release profile was subsequently plotted.

### 2.3 Cell Culture

Rat chondrocytes utilized in this study were obtained from Qingdao Sanjiu Biotechnology Co. Ltd. Following thawing, the cells were seeded at a density of 8 × 10^6^ cells per 10 cm culture dish and maintained in standard high‐glucose medium supplemented with 10% fetal bovine serum and 1% antibiotics. Upon reaching approximately 80% confluence, as observed microscopically, the cells were passaged. Passage 3 cells were ultimately employed for subsequent experimental procedures.

### 2.4 Biocompatibility and Biological Function of Cur‐MS

#### 2.4.1 Cytotoxicity and Vitality Assessment

To identify the cytotoxic and effective concentration range of Cur‐MS for subsequent experiments, cell viability was evaluated using the Cell Counting Kit‐8 (CCK‐8; Dojindo, Japan) following the manufacturer's instructions and established protocols. Briefly, chondrocytes were seeded in 96‐well plates and allowed to adhere. After 24 h, the cells were treated with Cur‐MS or Cur at the same dosage for 24–72 h. Subsequently, 10 μL of CCK‐8 reagent was added to each well and incubated for 2–4 h at 37°C. Absorbance was measured at 450 nm using a microplate reader. Chondrocytes were also cultured in 6‐well plates. After 24 h, the cells were treated with Cur‐MS for a duration of 72 h. Subsequently, cell viability was evaluated using a live/dead cell viability assay (Invitrogen, USA) in accordance with the manufacturer's protocol, followed by imaging with a confocal microscope (Nikon). Quantitative analysis of the number of live cells was performed using ImageJ software.

#### 2.4.2 The Effects of Cur‐MS on Apoptosis in Chondrocytes

A one‐step TUNEL apoptosis detection kit was employed to assess the effect of Cur‐MS on chondrocyte apoptosis. Chondrocytes were seeded onto coverslips in 24‐well plates at a density of 5 × 10^4^ cells/mL and subsequently treated with 10 ng/mL lipopolysaccharide (LPS) for 4 h. Following live/dead staining results, cells were exposed to high‐glucose medium containing different Cur‐MS for 24 h. Thereafter, cells were fixed with 4% paraformaldehyde for 15 min, permeabilized with 0.1% Triton X‐100 in PBS for 10 min, and blocked with 2% bovine serum albumin (BSA) in PBS for 45 min. The TUNEL detection solution was then prepared according to the manufacturer's protocol, and 100 μL was added to each well. Nuclear staining was performed using DAPI for 10 min. Fluorescence imaging was conducted using a confocal microscope.

#### 2.4.3 Immunofluorescence Staining of Chondrocyte

To examine the direct cellular effects of Cur‐MS, chondrocytes were seeded at a density of 5 × 10^4^ cells per well into 24‐well plates containing sterile cell crawling slides and allowed to adhere overnight in standard high‐glucose culture medium. Subsequently, the cells were randomly assigned to one of four experimental groups. The Control group received only the standard high‐glucose culture medium. The IL‐1β group was treated with an additional 10 ng/mL of IL‐1β. Cur‐MS were added to the culture medium at a concentration equivalent to 300 μM Cur, based on the total amount of Cur encapsulated within the microspheres. It is important to note that this concentration corresponds to the nominal equivalent dose of Cur encapsulated within the microspheres, rather than the actual free Cur concentration present in the culture medium. This distinction arises because the encapsulated drug is released gradually from the microspheres over time. The IL‐1β/Cur‐MS group was treated with both 10 ng/mL IL‐1β and Cur‐MS. Cells from all groups were harvested at two critical time points, 1 h post‐treatment to evaluate acute NF‐κB activation and 48 h post‐treatment to assess sustained inflammation and pro‐hypertrophic protein expression.

At each designated time point when cell coverslips were collected, immunofluorescence staining was conducted according to the following protocol. Initially, the cell coverslips were gently washed twice with PBS, followed by fixation in 4% paraformaldehyde at room temperature for 15 min. Subsequently, the coverslips were washed and permeabilized by incubation in PBS containing 0.2% Triton X‐100 for 10 min. Blocking was then performed using PBS supplemented with 5% BSA at room temperature for 1 h. The coverslips were incubated overnight at 4°C with primary antibodies diluted in 1% BSA/PBS, including rabbit anti‐NF‐κB p65, mouse anti‐iNOS, rabbit anti‐RUNX2, and mouse anti‐type X collagen (COL‐X). Following thorough washing with PBS, the samples were incubated with the appropriate Alexa Fluor‐conjugated secondary antibodies at room temperature for 1 h in the dark. Nuclear counterstaining was carried out using DAPI for 10 min. Finally, the coverslips were mounted onto slides with an anti‐fade mounting medium, and images were acquired using a confocal laser scanning microscope.

Following image acquisition, fluorescence images were analyzed using ImageJ software. In each field of view, the percentage of cells exhibiting clear nuclear localization of p65 was manually quantified. Subsequently, the average fluorescence intensity of iNOS, RUNX2, and COL‐X was measured to determine their relative expression levels. All immunofluorescence experiments were independently replicated a minimum of three times.

#### 2.4.4 ROS Assay

Seed chondrocytes into a 96‐well plate at a density of 1 × 10^4^ cells per well. Treat the cells according to the four groups previously described. On Day 3, aspirate the culture medium from each well and add 100 μL of DCFH‐DA working solution diluted in culture medium to achieve a final concentration of 10 μM per well. Incubate the plate at 37°C in a 5% CO_2_ atmosphere, protected from light, for 30 min. Following incubation, remove the probe‐containing solution and gently wash the cells two to three times with PBS to thoroughly eliminate any extracellular probe. Subsequently, add 100 μL of PBS to each well. Immediately measure the fluorescence intensity using a multifunctional microplate reader, setting the excitation wavelength to 485 nm and the emission wavelength to 525 nm, and record the fluorescence intensity for all wells. Furthermore, cells and supernatants were collected 24 h after treatment. Intracellular superoxide dismutase (SOD) activity was assessed using the WST‐1 assay. Malondialdehyde (MDA) levels in the cell supernatant were quantified employing the thiobarbituric acid (TBA) method.

#### 2.4.5 Western Blot

To assess the expression levels of key signaling pathway proteins and effector molecules, Western blot analysis was conducted on cell samples. Following appropriate treatment, cells were lysed on ice using RIPA buffer supplemented with protease and phosphatase inhibitors to extract total protein. Protein concentrations were quantified using the BCA assay. Equal amounts of protein were resolved by SDS‐PAGE and subsequently transferred onto PVDF membranes. The membranes were blocked at room temperature for 1 h with 5% non‐fat milk and then incubated overnight at 4°C with the following mouse‐derived primary antibodies: anti‐p65 (1:500, Santa Cruz), anti‐iNOS (1:500, Santa Cruz), anti‐RUNX2 (1:500, Santa Cruz), anti‐Collagen X (1:500, Abcam), and anti‐β‐actin (1:5000, Abcam). The following day, membranes were incubated with HRP‐conjugated secondary antibodies at room temperature for 1 h, developed and imaged using an ECL chemiluminescent substrate. The grayscale values of the bands were quantitatively analyzed using ImageJ. β‐actin served as the internal reference, and the relative expression level of the target protein was determined by calculating the ratio of its grayscale value to that of β‐actin.

### 2.5 Cartilage Pellet Formation

Chondrogenic differentiation was induced using a three‐dimensional pellet culture system, as previously described with modifications (Johnson et al. 2022). Resuspend the previously harvested passage 3 chondrocytes to a concentration of 2 × 10^5^ cells/mL. Subsequently, transfer 1 mL of the cell suspension into each 15 mL centrifuge tube and centrifuge at 1200 rpm for 5 min. Incubate all centrifuge tubes at 37°C in a humidified atmosphere containing 5% CO_2_. On the following day, carefully aspirate the supernatant and add 1 mL of high‐glucose chondrogenic induction medium supplemented with 100 nM dexamethasone, 50 μg/mL ascorbate‐2‐phosphate, 40 μg/mL proline, 100 μg/mL sodium pyruvate, 1 × ITS + Premix, and 10 ng/mL recombinant human TGF‐β1.

All samples were randomly assigned to four groups. The Control group was cultured with chondrogenic induction medium alone. The IL‐1β group received an additional 10 ng/mL of IL‐1β in the medium. The Cur‐MS group was supplemented with 300 μM of Cur, based on prior findings. The IL‐1β/Cur‐MS group was treated with both 10 ng/mL of IL‐1β and Cur‐MS. The corresponding media in the centrifuge tubes were replaced every 2 days according to the group assignments. All samples were collected on Day 14 for subsequent analyses. Concurrently, conduct live/dead staining on the samples from each group to evaluate the cellular viability within the specimens.

### 2.6 Treatment of Rat OA

A total of 32 6‐week‐old male SD rats, obtained from Beijing Vital River Laboratory Animal Technology Co. Ltd., were utilized in this study. The experimental protocol was approved by Qilu Hospital of Shandong University (Qingdao) Experimental Animal Ethics Committee and Qingdao Harwars Biotechnology Co. Ltd. Experimental Animal Ethics Committee. The approval number is AUP‐20250823‐001. Following a 7 days acclimation period under standard laboratory conditions, the rats were randomly assigned to four groups.

At the beginning of the experiment, all rats were anesthetized. Initially, 3% isoflurane was administered continuously by inhalation at a flow rate of 1 L/min. Once the rats' respiration stabilized, pentobarbital sodium was administered intraperitoneally at a dosage of 50 mg/kg. The control group received a single intra‐articular injection of 50 μL physiological saline into the right knee joint. The OA group was administered a 50 μL intra‐articular injection of 10 mg/mL sodium iodoacetate (MIA) solution to induce OA. For the Cur group, a single administration of 50 μL of Cur solution at a concentration of 300 μM was performed. Cur was dissolved in a 10 mg/mL solution of MIA to obtain a final drug concentration of 300 μM. Subsequently, 50 μL of this drug mixture was injected into the right knee joint of the rat. For the Cur‐MS group, Cur‐MS were suspended in 10 mg/mL MIA solution at a concentration of 5 × 10^6^ particles/mL, and 50 μL of this suspension was injected into the right knee joint cavity. Following injections, the joints of all animals were flexed and extended repeatedly for 15 min to facilitate thorough distribution of the injected solutions. Throughout the study, all rats were maintained under standard feeding conditions. On Day 29 post‐injection, all experimental rats were euthanized by intraperitoneal injection of sodium pentobarbital at a dosage of 150 mg/kg.

The efficacy of Cur‐MS treatment in OA rat models was assessed by quantifying the extent of joint swelling. Measurements of the tibial plateau width in both knee joints were taken on days 0, 14, and 28 using a vernier caliper. The initial measurement on Day 0 was designated as d0, while dT represented the width at each subsequent time point. The change in knee joint width (ΔW) was calculated by subtracting d0 from dT. The paw withdrawal threshold (PWT) was evaluated using a von Frey pain threshold detector to determine the pain sensitivity of the rats' hind paws. Rats were placed on a metal mesh platform and allowed to acclimate to the environment for 1 h. The experimental setting was maintained in a quiet condition to minimize disturbances. Appropriate von Frey filaments were gently applied from beneath the mesh to the plantar surface of the hind paws, and the minimum force eliciting a positive paw withdrawal response was recorded as the PWT. The test was conducted five times, with a two‐minute interval between trials to allow the animals to return to baseline. The average of these measurements was calculated and used as the final result.

### 2.7 Sample Testing

#### 2.7.1 Histology and Immunohistochemistry

The histological and immunohistochemical analyses were performed in accordance with established protocols from prior studies. Initially, the collected pellet and rats samples were washed with PBS and immediately fixed in 4% paraformaldehyde at 4°C for 24 h. Subsequently, the samples underwent dehydration through a graded ethanol series, clearing with xylene, and paraffin embedding. Tissue blocks were then sectioned continuously at a thickness of 5 μm using a microtome, and the resulting sections were mounted onto glass slides.

For hematoxylin and eosin (HE) staining, tissue sections were first dewaxed in xylene and subsequently rehydrated through a graded ethanol series. The sections were then stained with hematoxylin solution for 3–5 min. Following this, differentiation was carried out using acid alcohol, and bluing was achieved with ammonia water. The sections were counterstained with a 0.5% aqueous eosin solution. Finally, the sections underwent dehydration, clearing, and were mounted using neutral resin. To evaluate cartilage‐specific matrix synthesis, Safranin O (SO) staining was also conducted. Dewaxed and rehydrated sections were stained with a 0.1% aqueous SO solution for 10–15 min to specifically visualize glycosaminoglycans, followed by rapid dehydration, clearing, and mounting.

For immunohistochemical analysis, tissue sections were initially treated with 3% hydrogen peroxide to inhibit endogenous peroxidase activity, followed by blocking with 5% normal goat serum. Subsequently, the sections were incubated overnight at 4°C with primary antibodies, including rat anti COL‐II, MMP13, and CD68. After thorough washing with PBS, biotin‐conjugated secondary antibodies and horseradish peroxidase‐labeled streptavidin were applied sequentially. Color development was then achieved using a DAB staining kit. Finally, nuclei were counterstained with hematoxylin, and the sections were dehydrated, cleared, and mounted for microscopic examination.

The staining results were captured using an optical microscope equipped with a digital camera. Subsequently, ImageJ software was employed to quantify the percentage of positively stained areas in five randomly selected fields per sample, followed by statistical comparisons between groups.

#### 2.7.2 Quantitative Analysis

To analyze the matrix components within the cartilage pellets, the samples were initially subjected to uniform digestion. Total collagen content was quantitatively determined by measuring hydroxyproline levels. Samples underwent alkaline hydrolysis, and the resulting free hydroxyproline products were quantified. The measurement of free hydroxyproline was conducted following the method previously described (Wang et al. 2021). Using a collagen to hydroxyproline mass ratio of 7.25, the hydroxyproline content was subsequently converted to total collagen content.

Following the weighing of the collected pellet samples, they were incubated in a digestion buffer containing 1 mg/mL proteinase K and digested in a water bath shaker at 56°C for 8 h. Subsequently, the digested samples were centrifuged at 1600 rpm for 5 min, and the supernatant was collected for further analysis. GAG content, a critical marker for assessing cartilage formation, was determined via the 1,9‐dimethylmethylene blue (DMMB) dye‐binding assay. Equal volumes of the digested samples and standards were combined with freshly prepared DMMB dye in a 96‐well plate, and absorbance was immediately measured at 525 nm using a microplate reader. A standard curve was established using a series of chondroitin sulfate concentrations, and the GAG content in the samples was calculated accordingly.

To more precisely quantify the cartilage phenotype, COL‐II levels were measured using an enzyme‐linked immunosorbent assay (ELISA). A suitable volume of tissue digest was prepared and processed in accordance with the manufacturer's protocol for the rat COL‐II ELISA kit. Samples and a series of diluted standards were added to microplates pre‐coated with capture antibodies. Following incubation and washing steps, biotin‐labeled detection antibodies, streptavidin‐horseradish peroxidase conjugate, and substrate solution were sequentially applied. Upon termination of the enzymatic reaction, absorbance was measured at 450 nm. The absolute concentration of COL‐II in each sample was determined by reference to the standard curve.

To dynamically evaluate the catabolic activity of the cartilage matrix under inflammatory conditions, the release of sulfated glycosaminoglycans (sGAG) into the culture supernatant was monitored. At each medium change, conditioned media were collected and centrifuged at 12,000 rpm for 10 min at 4°C to remove cellular debris. The clarified supernatant was subsequently used to quantify sGAG concentration employing the previously described DMMB assay. Finally, the concentrations measured at various time points were integrated with the culture medium volume to calculate either the cumulative release or the daily average release rate, thereby providing a direct measure of cartilage matrix degradation in each experimental group.

#### 2.7.3 Real‐Time Fluorescent Quantitative Polymerase Chain Reaction (qPCR) Analysis

To pellets, the expression levels of cartilage‐related genes (SOX9, ACAN, and COL2A1), catabolic metabolism‐related genes (MMP13, iNOS, and COX‐2), and cartilage hypertrophy‐related genes (COL10A1 and RUNX2) were quantified by qPCR. To rat knee joint samples, COL2A1, MMP13, CD68, TNF‐α, IL‐6, and Arg‐1 were quantified by qPCR. Total RNA was extracted using TRIzol reagent (Invitrogen) and subsequently reverse transcribed with Moloney murine leukemia virus reverse transcriptase (Invitrogen). Gene expression was detected using the Fast Synergy Brands Green Master Kit in conjunction with the Light Cycler 480 system, following the manufacturers' protocols. Expression levels were normalized to glyceraldehyde 3‐phosphate dehydrogenase (GAPDH) as the internal reference gene. Data analysis employed the comparative threshold cycle (Ct) method, with relative gene expression calculated using the 2‐ΔΔCt formula; the average expression value of the control group served as the calibrator. All primer sequences are provided in Table 1.

**TABLE 1:** Primer sequences of related genes.

Gene	5′‐3′	Primer
SOX9	Forward	AAGAACAAGCCGCACGTCAA
	Reverse	CCGTTCTTCACCGACTTCCTC
ACAN	Forward	CAGAGGCAACCACAACAGACA
	Reverse	AGCTGGGAAGGCATAAGCATG
COL2A1	Forward	GCATTGCCTACCTGGACGAAG
	Reverse	TCACAGTCTCGCCCCACTTAC
MMP13	Forward	ACTGAGAGGCTCCGAGAAATG
	Reverse	GAACCCCGCATCTTGGCTT
iNOS	Forward	GGAGGAGCTGATGGAGTAGTAGCGG
	Reverse	CTACCTACCTGGGGAACACCTGGG
COX‐2	Forward	TAAGTGCGATTGTACCCGGAC
	Reverse	TTTGTAGCCATAGTCAGCATTGT
COL10A1	Forward	CCCTTTTTGCTGCTAGTATCC
	Reverse	CTGTTGTCCAGGTTTTCCTGGCAC
RUNX2	Forward	TGTTCTCTGATCGCCTCAGTG
	Reverse	CCTGGGATCTGTAATCTGACTCT
CD68	Forward	AGCCCAGATTCAGATGCGAGT
	Reverse	GATCCTGTTTGAATCCGAAGCT
TNF‐α	Forward	ATGGCCTCCCTCTCATCAGT
	Reverse	GCTTGGTGGTTTGCTACGAC
IL‐6	Forward	ACTTCCAGCCAGTTGCCTTCTTG
	Reverse	TGGTCTGTTGTGGGTGGTATCCTC
Arg‐1	Forward	AAAGGTCCCGCAGCATTAAG
	Reverse	TTGAAAGGGGCTGTCATTGG

### 2.8 Mining and Analysis of Publicly Available Transcriptome Data

To further validate the expression changes of key genes associated with chondrocyte phenotypes observed in the OA model employed in this study and to investigate their potential molecular mechanisms, we utilized the publicly available transcriptome dataset GSE114007, which comprises human knee articular cartilage tissue samples, obtained from the Gene Expression Omnibus (GEO) database of the National Center for Biotechnology Information (NCBI). Differential expression analysis was conducted using the GEO2R online tool integrated within the GEO platform. GEO2R performs statistical analyses based on the R packages limma and DESeq2. Within the GEO2R interface, samples were categorized with “OA” designated as the experimental group and “Normal” as the control group, ensuring accurate group assignments for all samples. The analysis was performed using thresholds of |log_2_ fold change| > 1 and an adjusted *p* < 0.05 to identify differentially expressed genes, resulting in a list of significant genes and a normalized expression matrix. From this dataset, key genes relevant to the current experimental study were extracted, including inflammation‐related genes (IL6, PTGS2 [encoding COX‐2], NOS2 [encoding iNOS], CD68, TNF‐α), genes involved in matrix synthesis and catabolism (COL2A1, ACAN, MMP13), chondrocyte hypertrophy‐associated genes (COL10A1, RUNX2), and the master regulatory gene of chondrogenic differentiation (SOX9).

### 2.9 Statistical Analysis

All experimental procedures were conducted in triplicate or more, and the results are expressed as the mean ± standard deviation. Statistical analyses and assessments of significance were carried out using GraphPad Prism software. Comparisons among multiple groups were conducted using one‐way analysis of variance (ANOVA), followed by pairwise comparisons employing Tukey's honestly significant difference test. Repeated measures data were analyzed via two‐way ANOVA with Bonferroni correction applied for multiple comparisons. Data that did not meet normality assumptions were analyzed using the Kruskal‐Wallis H test. A *p* < 0.05 was regarded as indicative of statistical significance.

## 3 Results

### 3.1 Cur‐MS at Various Concentrations Demonstrate Favorable Material Properties

**FIGURE 1:**
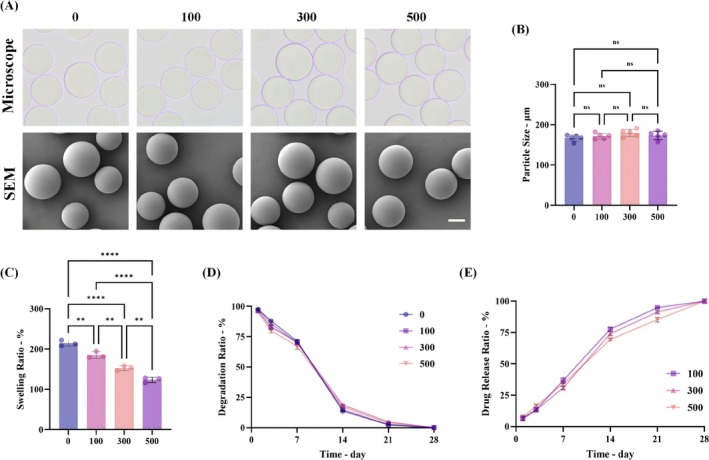
Material characterization of Cur‐MS in various concentrations. (A) Microscope and SEM of 0, 100, 300 and 500 μM of Cur in Cur‐MS. (B) Particle size of Cur‐MS. (C) Swelling ratio of Cur‐MS. (D) Degradation ratio of Cur‐MS. (E) Drug release ratio of Cur‐MS. Scale bar = 100 μm; ns indicates no statistical significance; **p < 0.01; ****p < 0.0001.

Under optical microscopy, the four groups of microspheres demonstrated uniform sizes (Figure 1A). Microspheres loaded with varying concentrations of Cur exhibited regular, circular morphologies. SEM analysis revealed that the GelMA microspheres retained their spherical shape following freeze‐drying, displaying a uniform and dense surface texture. Statistical analysis of particle size distribution showed no significant differences in average diameter among the four groups, with a narrow size distribution, indicating that the microspheres prepared via microfluidic control possessed high uniformity (Figure 1B).

Following rehydration in PBS, the freeze‐dried microspheres from all four experimental groups demonstrated excellent swelling behavior, indicative of a high water absorption capacity (Figure 1C). When incubated in PBS containing 1% type I collagenase, the microspheres from all groups exhibited degradation in a time‐dependent manner. The degradation process was initially slow during the first 7 days, followed by a gradual acceleration. By Day 28, complete degradation was observed in all groups, with no statistically significant differences among them (Figure 1D). The in vitro release profile revealed a sustained release of Cur from the microspheres. The release rate stabilized after 14 days and ceased by Day 28, coinciding with the complete degradation of the microspheres (Figure 1E). The strong correlation observed between the drug release profile and the microsphere degradation curve in this study confirms that drug release from Cur‐MS occurs via a combined mechanism involving both diffusion and degradation. These findings suggest that varying the concentration of Cur loading did not significantly alter the material properties of the GelMA microspheres.

### 3.2 Cur‐MS Mitigates Inflammation Induced Apoptosis, Inflammation, Oxidative Stress, and Hypertrophic Changes in Chondrocytes

**FIGURE 2:**
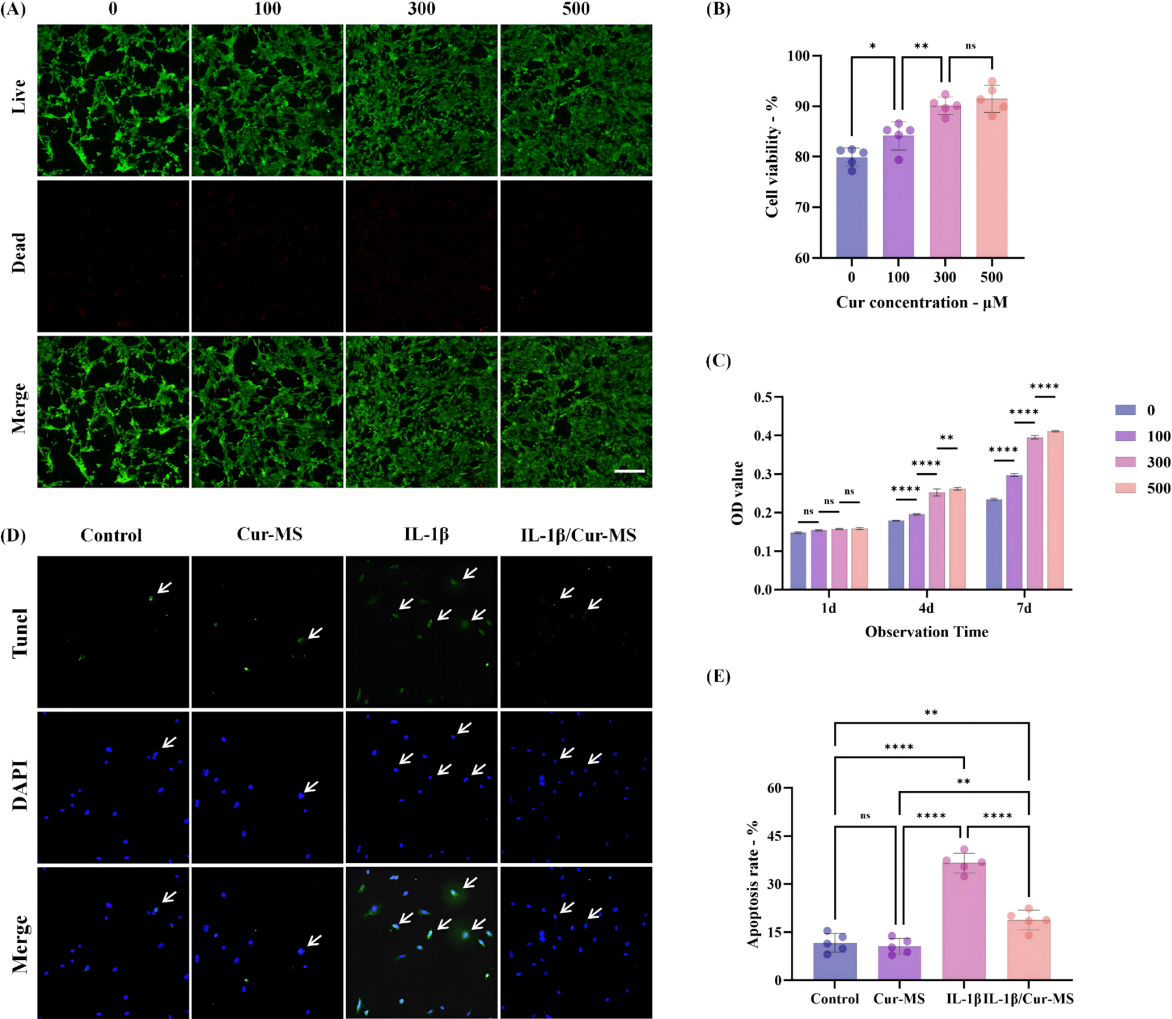
Effective concentration screening of Cur‐MS and its role in inhibiting chondrocyte apoptosis. (A) Following treatment, chondrocytes from each group were evaluated for cell viability using live/dead staining. Viable cells exhibited green fluorescence, whereas non‐viable cells displayed red fluorescence. (B) Quantitative analysis of the percentage of viable cells. (C) Assessment of the effects of varying concentrations of Cur on chondrocyte viability at 1, 4, and 7 days post‐treatment by the CCK‐8 assay. (D) TUNEL staining was employed to detect apoptosis in chondrocytes across each group. (E) Quantitative analysis of the percentage of positive TUNEL cells is presented. Scale bar = 100 μm; ns indicates no statistical significance; *p < 0.05; **p < 0.01; ****p < 0.0001.

To assess the direct protective effects of Cur‐MS at the cellular level, we initially performed in vitro cell experiments. Live/dead staining results demonstrated that compared to an equivalent dose of free Cur, the Cur‐MS treatment group demonstrated a higher cell survival rate (Figure S1). The results demonstrate that encapsulation within GelMA microspheres significantly enhances the cytoprotective efficacy of Cur. Cur concentrations of 300 and 500 μM in Cur‐MS produced comparable effects in promoting chondrocyte proliferation (Figure 2A). Additionally, concentration screening experiments using the CCK8 assay revealed that Cur, within the 0–500 μM range, did not exhibit significant cytotoxicity toward chondrocyte viability (Figure 2B,C). Based on these findings, Cur‐MS with a concentration of 300 μM Cur was selected for all subsequent experiments because it reached the maximum effect plateau and higher concentrations did not show additional benefits.

To examine the effect of Cur‐MS on IL‐1β‐induced apoptosis in chondrocytes, we conducted TUNEL staining analysis. As illustrated in Figure 2D, stimulation with IL‐1β significantly increased the number of TUNEL‐positive cells, indicating that the inflammatory environment induced substantial apoptosis. In contrast, the Cur‐MS group exhibited no evident apoptotic cells, comparable to the control group. Furthermore, the IL‐1β/Cur‐MS group demonstrated a marked reduction in TUNEL‐positive signals. Quantitative analysis confirmed that the percentage of apoptotic cells in the IL‐1β group was significantly higher than that in the control group, whereas the Cur‐MS group significantly decreased the apoptosis rate (Figure 2E).

**FIGURE 3:**
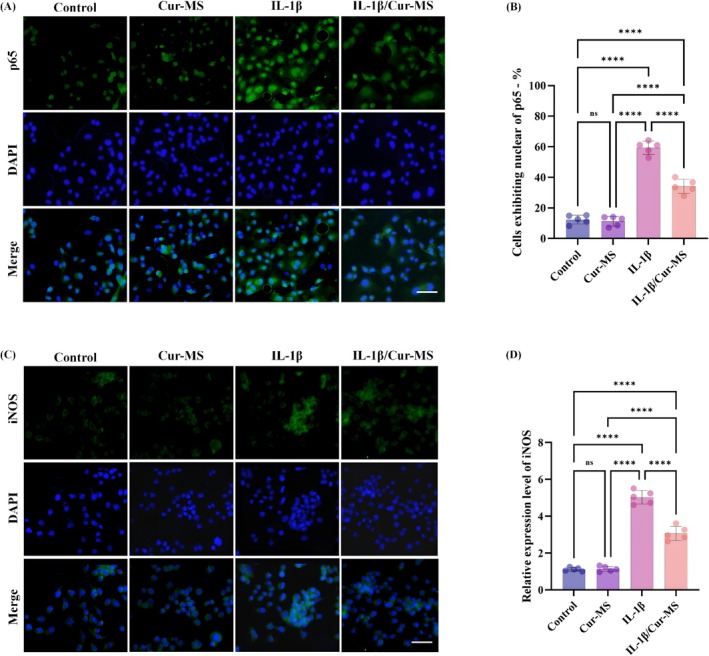
Cur‐GelMA inhibits IL‐1β‐Induced inflammatory response in chondrocytes. (A) Immunofluorescence staining illustrating the subcellular localization and nuclear translocation of NF‐κB p65. (B) Quantitative analysis of the percentage of cells exhibiting nuclear localization of p65. (C) Immunofluorescence staining of iNOS. (D) Quantification of the average fluorescence intensity of iNOS. Scale bar = 50 μm; ns indicates no statistical significance; ****p < 0.0001.

**FIGURE 4:**
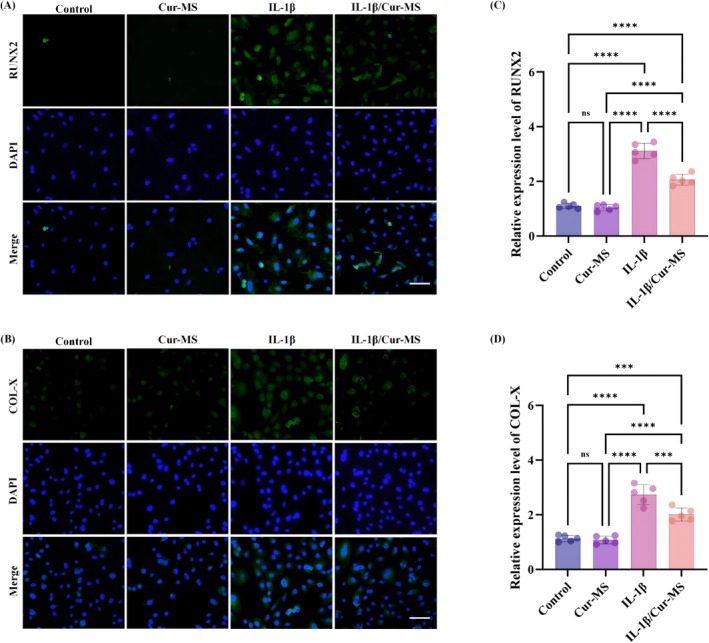
Cur‐MS inhibits IL‐1β‐Induced chondrocyte hypertrophy and oxidative stress. (A, B) Immunofluorescence staining and quantification of RUNX2. (C, D) Immunofluorescence staining and quantification of COL‐X. Scale bar = 50 μm; ns indicates no statistical significance; ***p < 0.001; ****p < 0.0001.

To elucidate the cellular mechanisms underlying the anti‐inflammatory and anti‐hypertrophic effects of Cur‐MS, we investigated the expression and subcellular localization of key proteins via immunofluorescence staining. In the context of the inflammatory response, a significant nuclear translocation of the NF‐κB p65 protein was observed following 1 h of IL‐1β stimulation, as evidenced by the co‐localization of green fluorescence with blue DAPI nuclear staining (Figure 3A). Pretreatment with Cur‐MS effectively inhibited this translocation, resulting in the retention of p65 predominantly within the cytoplasm. Quantitative analysis of the proportion of p65 nuclear‐positive cells corroborated this inhibitory effect (Figure 3B). Furthermore, after 24 h of IL‐1β stimulation, the expression of the inflammatory mediator iNOS was markedly upregulated, as indicated by increased green fluorescence intensity (Figure 3C). Co‐treatment with Cur‐MS significantly attenuated iNOS protein expression (Figure 3D). Regarding hypertrophic tendencies, stimulation with IL‐1β also upregulated the expression of the key transcription factor RUNX2 and COL‐X, promoting their accumulation within the cell nucleus (Figure 4A–D). These findings confirm that Cur‐MS can concurrently inhibit IL‐1β‐induced inflammatory activation and pro‐hypertrophic differentiation signaling pathways.

**FIGURE 5:**
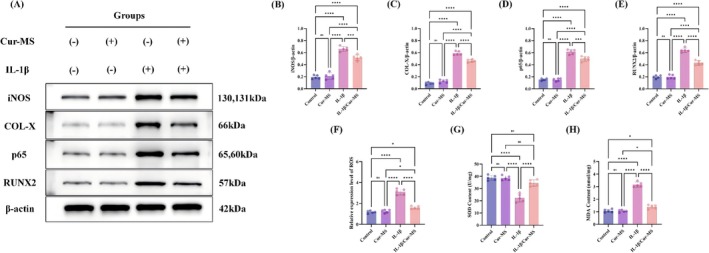
Western blot analysis of inflammation and hypertrophy related proteins. (A) Protein blot bands corresponding to each experimental group. (B–E) Relative expression levels of various proteins. (F) Relative quantitative analysis of intracellular ROS levels detected. (G, H) The SOD and MDA content of each experimental group. Ns indicates no statistical significance; *p < 0.05; ***p < 0.001; ****p < 0.0001.

To assess the regulatory effects of Cur‐MS on inflammation and hypertrophy‐related pathways at the protein level, western blot analysis was employed to measure the expression levels of p65, iNOS, RUNX2, and COL‐X in each cell group. As illustrated in Figure 5A, stimulation with IL‐1β significantly increased the phosphorylation of p65, indicating activation of the NF‐κB signaling pathway. Concurrently, the protein expression levels of iNOS, RUNX2, and COL‐X were markedly elevated. Treatment with Cur‐MS effectively inhibited p65 activation and significantly reduced the protein expression levels of iNOS, RUNX2, and COL‐X. Quantitative analysis of grayscale values demonstrated that, compared to the IL‐1β group, the relative protein expression levels of all targets in the IL‐1β/Cur‐MS group were significantly decreased (Figure 5B–E). These findings suggest that Cur‐MS effectively suppresses IL‐1β‐induced NF‐κB pathway activation, inflammatory responses, and hypertrophic progression at the protein level, corroborating the results obtained from immunofluorescence staining.

Oxidative stress plays a critical role in inflammatory injury. Intracellular ROS levels were assessed using the DCFH‐DA fluorescent probe, revealing that stimulation with IL‐1β induced a pronounced increase in ROS within chondrocytes, as evidenced by a significant elevation in fluorescence intensity (Figure 5F). Conversely, the ROS level in the IL‐1β/Cur‐MS group was significantly reduced compared to that in the IL‐1β group, indicating a marked suppression of intracellular ROS production. Compared to the control group, IL‐1β stimulation resulted in a significant reduction in SOD activity within chondrocytes, indicating a marked impairment of the cells' endogenous antioxidant defense mechanisms. Concurrently, MDA levels were significantly elevated, suggesting an increase in lipid peroxidation. Pretreatment with Cur‐MS significantly restored SOD activity to near‐normal levels and markedly reduced MDA content relative to the IL‐1β group. In contrast, the Cur group exhibited no significant differences in SOD activity or MDA content compared to the control group, indicating that Cur alone does not exert oxidative toxicity on chondrocytes. These findings demonstrate that Cur‐MS effectively mitigates IL‐1β‐induced oxidative imbalance by enhancing endogenous antioxidant enzyme activity and decreasing lipid peroxidation. These results align with the ROS detection data, collectively confirming the protective effect of Cur‐MS against oxidative stress‐induced damage in chondrocytes.

In summary, at the cellular level, Cur‐MS exhibits multiple protective effects. It effectively inhibits IL‐1β‐induced chondrocyte apoptosis, suppresses the activation of the NF‐κB inflammatory signaling pathway and the expression of downstream iNOS, mitigates oxidative stress, and inhibits the activation of hypertrophy‐promoting transcription factors RUNX2 and COL‐X. These findings provide a cellular basis for the protective effects observed subsequently in cartilage pellet tissue.

### 3.3 Cur‐MS Protects Cartilage Pellets From Inflammation Induced Matrix Degradation and Phenotypic Alterations

**FIGURE 6:**
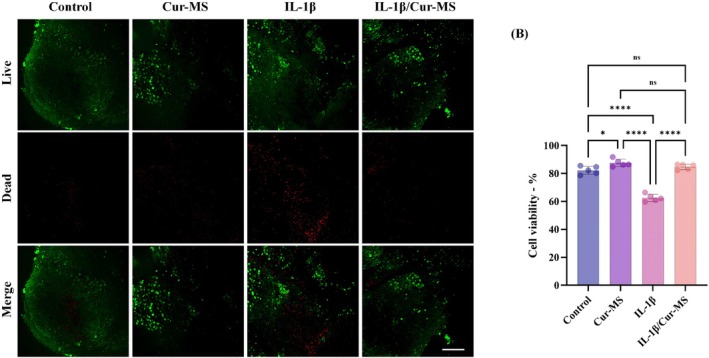
Protective effects of Cur‐MS on the viability of cartilage pellet cultures. (A) Representative images of live/dead staining of cartilage pellets from each group following cultivation. (B) Quantitative analysis of the proportion of live cells. Scale bar = 100 μm; ns indicates no statistical significance; *p < 0.05; ****p < 0.0001.

Building upon the verification of cellular‐level functions, we further assessed the effects of Cur‐MS on cartilage pellets. As illustrated in Figure 6A, nearly all cells within the control group pellets exhibited green fluorescence that was indicative of viable cells, with only occasional dead cells observed in the central region of the tissue. This finding demonstrates that the three‐dimensional culture system effectively maintained high cell viability. In contrast, the IL‐1β group displayed substantial cell death, evidenced by a pronounced increase in red fluorescence signals, particularly large clusters of dead cells localized in the pellet center. Notably, the inclusion of Cur‐MS during co‐culture with IL‐1β markedly mitigated this effect, significantly decreasing the number of dead cells. Quantitative analysis of the proportion of live cells corroborated these observations, revealing that the live cell rate in the IL‐1β group was significantly reduced compared to the control group, whereas Cur‐MS restored cell viability (Figure 6B).

**FIGURE 7:**
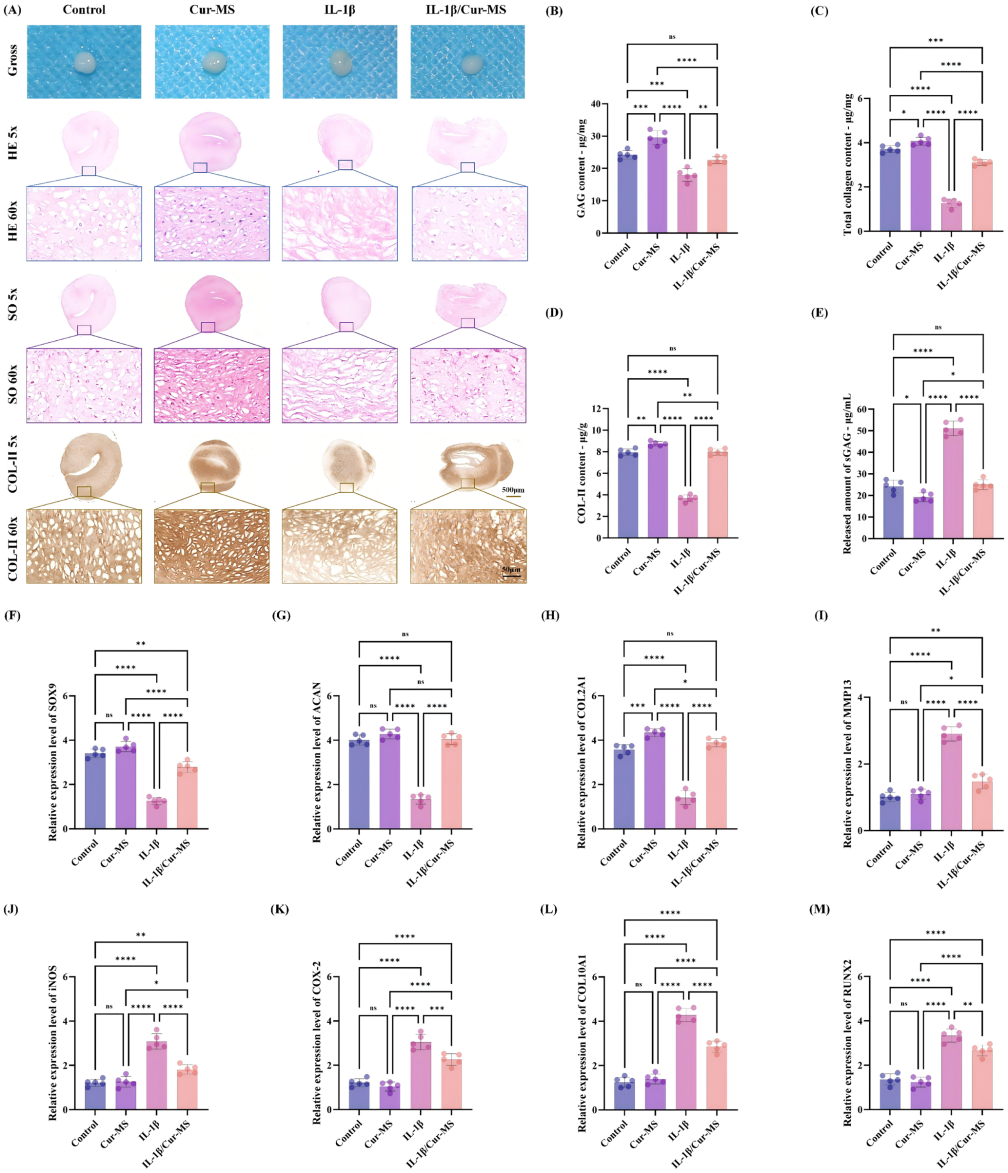
The effects of Cur‐MS on the regeneration of cartilage pellets. (A) HE, SO, and COL‐II staining was employed to examine the overall tissue architecture of the pellet and distribution of GAG and COL‐II. (B–E) Biochemical quantitative analysis of Cur‐MS effects on cartilage pellet matrix synthesis and degradation. (F–M) QPCR analysis of Cur‐MS on cartilage pellet in gene expression. Ns indicates no statistical significance; *p < 0.05; **p < 0.01; ***p < 0.001; ****p < 0.0001.

In histological staining (Figure 7A), HE staining revealed that all pellet groups preserved their dense structure. Compared to the control and Cur‐MS groups, which displayed well‐defined cartilage lacunae structures that the IL‐1β group lacked, these characteristic cartilage features were absent. Notably, the IL‐1β/Cur‐MS group exhibited a marked restoration of cartilage architecture. However, notable differences were evident in SO staining. Treatment with IL‐1β induced a marked depletion of the cartilage‐specific GAG component, as evidenced by markedly diminished staining intensity in the central region of the tissue. Conversely, the IL‐1β/Cur‐MS group significantly preserved GAG content, with staining intensity and distribution closely resembling those observed in the control group. Similarly, immunohistochemical staining for COL‐II demonstrated a substantial decrease in the positively stained area within the IL‐1β group, while the Cur co‐treatment group exhibited enhanced and more uniform COL‐II deposition.

To corroborate the histological findings, we performed additional quantitative analyses on cartilage pellet samples. Measurements of the digested pellet tissue revealed that IL‐1β treatment significantly decreased the content of GAG, total collagen, and COL‐II (Figure 7B–D). The Cur‐MS effectively counteracted these reductions, resulting in a significant increase in all content. Furthermore, we evaluated the catabolic activity of the cartilage‐specific matrix by quantifying the release of sGAG into the conditioned medium (Figure 7E). The data indicated that IL‐1β stimulation substantially elevated cumulative sGAG release, reflecting enhanced ECM degradation. The IL‐1β/Cur‐MS group significantly attenuated this effect. Collectively, these quantitative results demonstrate that Cur‐MS promotes the synthesis and retention of cartilage‐specific matrix components at the tissue level while mitigating the excessive catabolic activity induced by IL‐1β.

To elucidate the molecular mechanisms underlying the observed phenotypic alterations, we analyzed the expression profiles of key genes using qPCR. Concerning anabolic metabolism, the IL‐1β group exhibited downregulation of the core cartilage gene SOX9 and its downstream targets ACAN and COL2A1 (Figure 7F–H). Notably, IL‐1β/Cur‐MS significantly counteracted this inhibitory effect, restoring SOX9 and ACAN transcription levels to values comparable to those of the control group and partially recovering COL2A1 expression. Regarding catabolic metabolism and inflammatory responses, IL‐1β markedly induced the expression of the matrix‐degrading enzyme MMP13, as well as the inflammatory mediators iNOS and COX‐2 (Figure 7I–K). Treatment with IL‐1β/Cur‐MS substantially suppressed the upregulation of these genes, with particularly pronounced inhibition observed for MMP13 and iNOS. In terms of hypertrophy‐associated genes, IL‐1β stimulation significantly elevated mRNA levels of COL10A1 and RUNX2 (Figure 7L,M). The IL‐1β/Cur‐MS group effectively inhibited this upregulation, indicating that Cur‐MS impedes IL‐1β‐induced hypertrophic differentiation at the transcriptional level. Collectively, these gene expression data align with our protein and phenotypic findings, demonstrating at the molecular level that Cur‐MS preserves chondrocyte homeostasis by coordinately regulating anabolic and catabolic metabolism, suppressing inflammation, and modulating hypertrophy‐related gene networks.

### 3.4 Cur‐MS Inhibits the Progression of OA by Preserving Cartilage Integrity and Mitigating Inflammatory Responses

**FIGURE 8:**
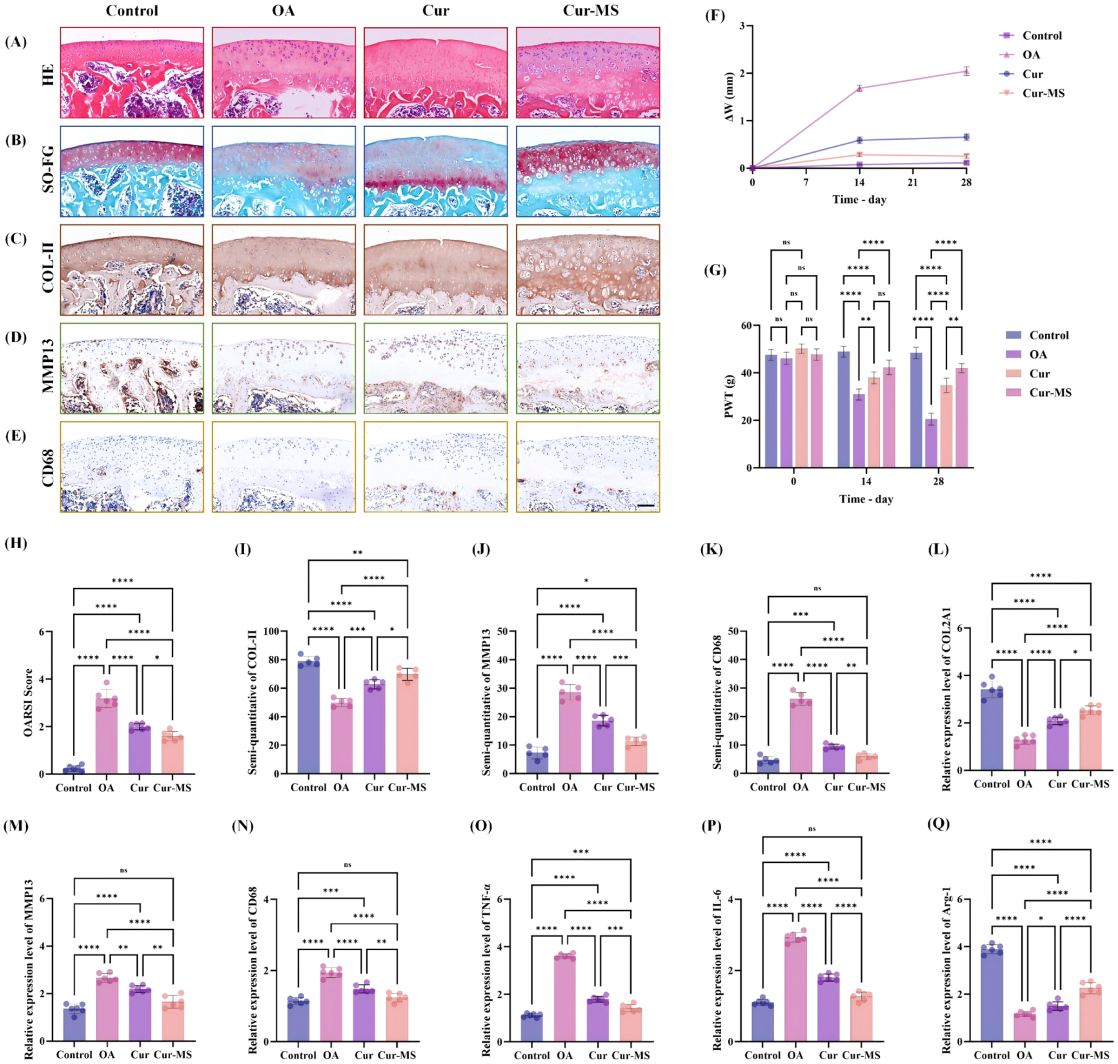
Immunomodulatory and cartilage protective effects of Cur‐MS on the immune microenvironment in a rat model of OA. (A–E) HE, SO and immunohistochemical staining of COL‐II, MMP13 and CD68. (F) The change in knee joint width of each group. (G) PWT results of each group. (H) OARSI score of each group. (I–K) Semi‐quantitative of COL‐II, MMP13 and CD68. (L–Q) QPCR analysis of each group in gene expression. Scale bar = 100 μm; ns indicates no statistical significance; *p < 0.05; **p < 0.01; ***p < 0.001; ****p < 0.0001.

Following MIA injection, the OA group exhibited significant joint swelling, as evidenced by a continuous increase in the joint swelling index. Concurrently, the PWT on the affected side was markedly reduced. In contrast, the Cur‐MS group demonstrated a consistently lower joint swelling index from the time of modeling through Day 28, approximating values observed in the Control group but lower than the Cur group. Furthermore, the PWT in the Cur‐MS group was significantly elevated compared to that in the Cur and OA groups (Figure 8F,G). These findings suggest that intra‐articular administration of Cur‐MS effectively inhibits joint swelling and substantially enhances the mechanical pain threshold. Cur‐MS can demonstrate enhanced therapeutic effects owing to their superior sustained release properties compared to free Cur. Consequently, Cur‐MS exhibit potent anti‐inflammatory and analgesic properties and can significantly improve joint function in OA rat models.

Knee joint samples were collected 4 weeks post‐modeling and treatment to facilitate the evaluation of experimental outcomes related to OA prevention. Initially, histological analyses were performed to assess pathological alterations in the articular cartilage. HE and SO‐FG staining revealed that cartilage lacuna in the OA group exhibited disorganization, near‐complete loss of proteoglycans, and severe degradation of the cartilage matrix. In contrast, the Cur‐MS and Control groups demonstrated well‐defined chondrocytes, orderly arranged cartilage lacuna, and abundant cartilage matrix (Figure 8A,B). OARSI scoring indicated that although the Cur‐MS group's scores differed from those of the Control group, they were significantly lower than those observed in the Cur and OA group (Figure 8H). Immunohistochemical staining was employed to evaluate the expression levels of COL‐II, MMP13, and CD68 within the articular cartilage (Figure 8C–E). COL‐II positivity was markedly reduced in the OA group compared to the Cur‐MS group, while MMP13 expression exhibited a similar pattern consistent with the aforementioned staining results. Furthermore, CD68 expression was significantly elevated in the OA group relative to the Cur‐MS group. Furthermore, the semi‐quantitative analysis of immunohistochemistry corroborates the aforementioned conclusion (Figure 8I–K).

To assess the regulatory effects of Cur‐MS on cartilage metabolism and the inflammatory microenvironment in vivo, qPCR was employed to measure the mRNA expression levels of COL2A1, MMP13, CD68, and the pro‐inflammatory cytokines TNF‐α and IL‐6 in the articular cartilage of rats from each experimental group (Figure 8L–Q). Compared to the Control group, the mRNA expression level of COL2A1 in the articular cartilage of rats in the OA group was significantly reduced, indicating an inhibition of cartilage matrix synthesis. Concurrently, the expression levels of MMP13, CD68, TNF‐α, and IL‐6 were markedly elevated, suggesting pronounced activation of matrix catabolism, macrophage infiltration, and inflammatory responses within the OA joint. Treatment with free Cur and Cur‐MS significantly reversed these alterations relative to the OA group. Specifically, COL2A1 and Arg‐1 expression was substantially increased, while the mRNA levels of MMP13, CD68, TNF‐α, and IL‐6 were significantly suppressed. Certainly, the impact of free Cur on related factors is considerably less pronounced than that of Cur‐MS. In summary, intra‐articular administration of Cur‐MS effectively upregulates COL2A1 expression in OA joints, inhibits MMP13‐mediated matrix degradation, and reduces CD68‐positive macrophage infiltration as well as inflammatory cytokine levels including TNF‐α and IL‐6. These findings demonstrate that Cur‐MS confer a protective effect by modulating cartilage metabolism and the inflammatory microenvironment.

### 3.5 Transcriptomic Characteristics of OA Cartilage Compared to Normal Cartilage in Public Transcriptome Datasets

Utilizing the publicly available transcriptome dataset GSE114007, a systematic comparison was performed to examine genome‐wide expression differences between OA cartilage and normal cartilage. A total of 2182 differentially expressed genes were identified, comprising 1308 upregulated and 874 downregulated genes in the OA group relative to the normal group.

**FIGURE 9:**
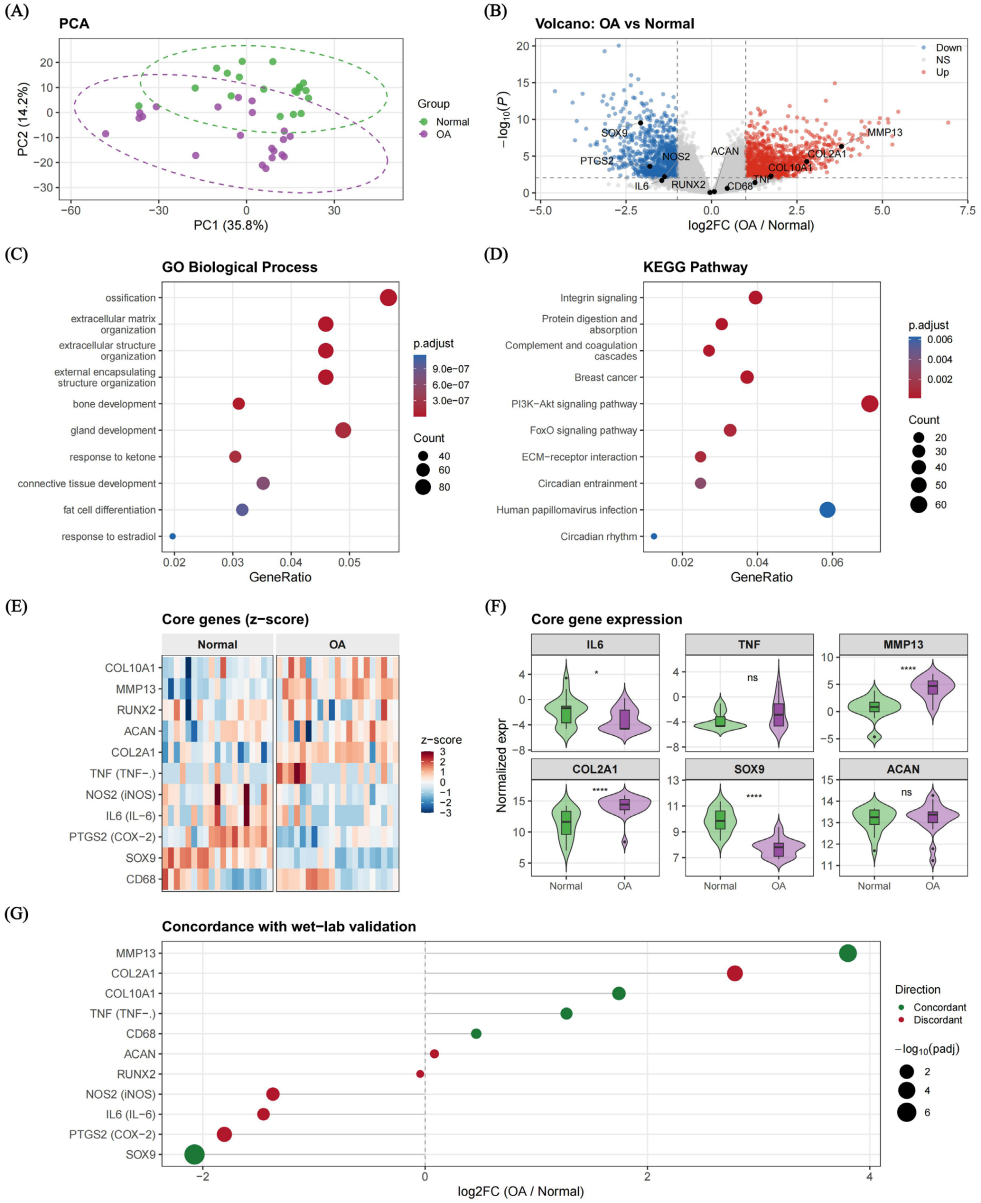
A transcriptomic overview of OA and normal knee joint cartilage (GSE114007) is presented. (A) Principal component analysis was conducted using highly variable genes. The normal group depicted in green and the OA group in purple, dashed lines represent confidence ellipses for each group. (B) A volcano plot illustrates differential gene expression. Red and blue denote significantly upregulated and downregulated genes, respectively. Gray indicates non‐significant genes and black dots highlight core genes. (C) GO biological process enrichment is displayed by a bubble plot. (D) KEGG pathway enrichment is similarly represented by a bubble plot. (E) A heatmap shows the *z*‐score normalized expression of 11 core genes. (F) Violin and box plots depict the expression levels of six core genes. (G) A comparison between bioinformatics results and expected expression directions from wet lab experiments. Green indicating concordance and red indicating discordance, dot size corresponds to the −log10 adjusted *p*‐value. Differential gene expression was defined by |log2 fold change| ≥ 1 and *p*adj < 0.05, identifying 1308 upregulated and 874 downregulated genes.

Principal component analysis (PC1: 35.8%, PC2: 14.2%) demonstrated that OA and normal groups could be differentiated along the principal component plane, although some overlap was observed. This finding indicates that while overall transcriptomic differences are evident, they are modulated by individual heterogeneity (Figure 9A). The differential expression volcano plot (Figure 9B) revealed significant upregulation of MMP13 and COL10A1, alongside significant downregulation of SOX9 in OA cartilage. Inflammation‐related molecules PTGS2 and NOS2 exhibited mild downregulation, whereas IL6, TNF, and CD68 showed no significant alterations. GO enrichment analysis (Figure 9C) identified significant enrichment in biological processes including ossification and extracellular matrix organization. Kyoto Encyclopedia of Genes and Genomes (KEGG) pathway analysis (Figure 9D) indicated enrichment in the PI3K‐Akt signaling pathway, ECM‐receptor interaction, and complement cascade pathways. The core gene heatmap (Figure 9E) and quantitative comparisons (Figure 9F) confirmed a significant increase of MMP13 in the OA group, a significant decrease of SOX9, a paradoxical increase of COL2A1, a mild decrease of IL6, and no significant differences in TNF and ACAN expression. Directional consistency analysis (Figure 9G) demonstrated that the upregulation of MMP13, COL10A1, TNF, and CD68, as well as the downregulation of SOX9, aligned with expectations from wet laboratory experiments. In contrast, expression changes in COL2A1, ACAN, IL6, PTGS2, NOS2, and RUNX2 exhibited some inconsistencies. These findings suggest that transcriptomic characteristics derived from cartilage tissue differ from those observed in synovium or cell models, underscoring the necessity for tissue‐specific interpretation. Collectively, public data corroborate the core molecular imbalance of MMP13 and SOX9 in OA cartilage, thereby providing external validation for the cartilage‐protective targets identified by Cur‐MS.

## 4 Discussion

OA is a chronic inflammatory disorder primarily characterized by the progressive degeneration of articular cartilage. Its pathogenesis is multifactorial, involving immune responses, inflammation, oxidative stress imbalance, and ECM metabolic dysregulation. Despite recent advances in therapeutic approaches, there remains a paucity of disease‐modifying agents capable of effectively delaying cartilage degradation in clinical settings. Cur, a natural polyphenolic compound, has garnered considerable interest due to its multi‐targeted anti‐inflammatory, antioxidant, and cartilage protective properties. However, its clinical application is hindered by poor aqueous solubility, rapid metabolism in vivo, and limited bioavailability. To overcome these limitations, the present study developed a GelMA hydrogel microsphere delivery system encapsulating Cur. Furthermore, we employed an innovative, multi‐tiered evaluation strategy encompassing 2D cellular mechanistic studies, 3D tissue biomimetic validation, and assessments in animal models in vivo. This comprehensive approach systematically elucidated the anti‐inflammatory and cartilage‐protective effects of Cur‐MS across various experimental models for the first time. The findings demonstrated that Cur‐MS effectively inhibited chondrocyte apoptosis, inflammatory responses, oxidative stress, and hypertrophic differentiation at both cellular and microtissue levels. In a rat model of knee OA, a single intra‐articular injection of Cur‐MS significantly improved the degree of cartilage degeneration and restored the metabolic balance of cartilage‐specific matrix synthesis. Notably, Cur‐MS also remodeled the inflammatory microenvironment within the joint cavity, thereby impeding OA progression. These results not only substantiate the therapeutic potential of Cur‐MS for OA but also confirm the protective effects of Cur through a robust multi‐model experimental design, thereby providing a solid theoretical foundation for its clinical translation.

During the preparation and characterization of Cur‐MS, no significant differences were observed in particle size distribution or in vitro degradation behavior among Cur‐MS formulations with varying drug loading concentrations. Although this lack of a dose‐dependent effect may be considered a negative result, it holds considerable pharmaceutical significance. Firstly, it suggests that within the investigated range of drug loading concentrations, Cur incorporation did not induce detectable structural disruption to the crosslinked network of GelMA microspheres, thereby preserving the physicochemical stability of the microsphere matrix. This observation aligns with previous studies on GelMA microsphere delivery systems, which have shown that GelMA concentration and photoinitiated crosslinking density predominantly govern microsphere particle size and degradation characteristics, while moderate variations in drug loading exert minimal influence on these fundamental parameters. Secondly, the uniformity in particle size and degradation behavior across different drug loading groups provides a critical basis for attributing the concentration‐dependent biological effects observed in subsequent experiments primarily to differences in Cur release profiles, rather than to confounding variations in the physical properties of the microsphere carriers. From a formulation development perspective, these findings also underscore the robustness of the Cur‐MS preparation process, thereby establishing a solid foundation for quality control in future scale‐up production and clinical translation efforts.

The protective effects observed in this study were initially and systematically validated at the cellular level. IL‐1β, recognized as a pivotal pro‐inflammatory cytokine in the pathogenesis of OA, activates the NF‐κB signaling pathway, thereby initiating a cascade of downstream inflammatory responses and catabolic processes. This pathway is widely regarded as a central driver of cartilage degeneration. In the present study, chondrocytes exposed to IL‐1β exhibited a significant increase in apoptosis, as demonstrated by TUNEL staining, accompanied by pronounced nuclear translocation of NF‐κB p65. Concurrently, both gene and protein expression levels of inflammatory mediators iNOS and COX‐2 were significantly upregulated, and oxidative stress levels were markedly upregulated. Notably, treatment with Cur‐MS effectively reversed these pathological changes. Furthermore, the expression of the pro‐hypertrophic transcription factor RUNX2 was significantly induced by IL‐1β, whereas Cur‐MS treatment substantially downregulated RUNX2 expression. Collectively, these results indicate that Cur synergistically inhibits NF‐κB‐mediated inflammatory cascades, ROS induced oxidative damage and prevents nuclear accumulation of pro‐hypertrophic transcription factors. This study thus delineates a multi‐targeted cellular protective mechanism, providing molecular‐level theoretical support for the protective role of Cur‐MS in cartilage microtissue.

A principal innovation of this study is the transition from a chondrocyte monolayer model to a cartilage microtissue model. While two‐dimensional monolayer cultures offer convenience, they fail to accurately replicate the three‐dimensional collagen network microenvironment in which chondrocytes naturally reside in vivo. Within this three‐dimensional cartilage microenvironment, cell‐matrix interactions, as well as ECM deposition and remodeling processes, differ markedly from those observed under physiological conditions. Pellet culture, widely recognized as an in vitro model for cartilage tissue engineering, involves the formation of dense spherical aggregates through high‐density centrifugation of cells. The patterns of ECM synthesis and deposition in these pellets more closely resemble the cartilage development process in vivo. In the present study, stimulation with IL‐1β resulted in a significant reduction in SO staining intensity within pellets and a substantial decrease in the immunohistochemically positive area for COL‐II. Biochemical quantification of GAG and total collagen further confirmed a pronounced loss of matrix components. Moreover, the cumulative release of sGAG in the culture medium was markedly elevated, indicating excessive activation of catabolic metabolism within the pellets. Gene expression analyses related to cartilage matrix synthesis, inflammation and hypertrophy corroborated these findings. Treatment with Cur‐MS significantly reversed these alterations, restoring the content of major ECM components and reducing sGAG release. These results demonstrate that Cur‐MS retain their cartilage protective function within a three‐dimensional biomimetic system. Collectively, these findings validate that Cur‐MS can counteract inflammatory insults and maintain ECM homeostasis in a cartilage microtissue model that more closely approximates the physiological environment. Besides, our analysis indicates that the primary cause of the hollow phenomenon observed in the pellets is the discrepancy between the size of the spherical cell aggregates and the limitations imposed by diffusion. Histological and immunohistochemical staining in the experimental group demonstrated that Cur‐MS effectively inhibits IL‐1β‐induced inflammation and apoptosis. However, nutrient and oxygen delivery relies on passive diffusion through the extracellular matrix rather than vascular transport. When the pellet diameter exceeds a critical threshold, oxygen and nutrients cannot adequately reach the central region. Consequently, despite the drug's efficacy in inhibiting apoptosis, central cells are deprived of essential physical resources required for survival, leading to necrosis and cavity formation. The drug's protective effect on peripheral cells promotes more active synthesis of cartilage matrix, thereby enhancing evaluation metrics such as quantitative analysis. It is noteworthy that, within cartilage microtissues, the extent of expression recovery for ACAN and SOX9 surpasses that of COL2A1. This disparity may hold biological significance. As the principal transcription factor governing chondrogenic differentiation, the restoration of SOX9 establishes the foundation for the activation of downstream anabolic genes. In contrast, COL2A1 expression is co‐regulated by multiple signaling pathways, including TGF‐β/Smad and Wnt/β‐catenin, and its complete recovery may necessitate a more intricate reconstitution of these networks. Consequently, the efficient short‐term repair of ACAN by Cur‐MS likely reflects its selective influence on SOX9‐dependent pathways. The delayed recovery of COL2A1 implies that the synthesis and reconstruction of ECM structural proteins require an extended temporal window. This observation offers a novel perspective for the detailed investigation of Cur‐MS's mechanism of action, suggesting that it preferentially restores the primary anabolic program, whereas ECM structural reconstruction constitutes a subsequent phase.

The most critical evidence in this study derives from validation using an in vivo rat model of knee OA. MIA rapidly induces cartilage metabolic dysfunction and chondrocyte death by inhibiting glutamine synthetase, thereby serving as a well‐established and efficient single‐injection chemically induced OA model. Joint swelling represents a direct external indicator of synovial inflammation and joint effusion. Our findings demonstrated that joint swelling emerged and progressively intensified in the OA group following MIA injection, confirming that MIA effectively induced an acute synovial inflammatory response. In contrast, the Cur‐MS group exhibited consistently low levels of joint swelling, which corresponded with decreased infiltration of CD68‐positive macrophages in the synovial tissue and downregulated expression of pro‐inflammatory cytokines. These results suggest that Cur‐MS mitigate increases in synovial vascular permeability and inflammatory exudation by inhibiting the intra‐articular inflammatory cascade, thereby resulting in a macroscopic reduction of joint swelling. The PWT is a well‐established behavioral measure for evaluating OA‐related pain. The pathogenesis of pain in the MIA model is multifaceted, involving direct chondrocyte damage that leads to local metabolite accumulation and persistent inflammation, which in turn induces peripheral nerve sensitization and central sensitization. Our findings demonstrated a significant reduction in PWT in the OA group, indicative of mechanical allodynia. Conversely, the PWT in the Cur‐MS group approximated normal levels. This effect can be attributed to the sustained local release of Cur from Cur‐MS, which inhibits the production of inflammatory mediators and diminishes the release of pain‐induced factors. Furthermore, Cur exhibits neuroprotective properties that may mitigate central sensitization through modulation of microglial activation. Additionally, the observed reduction in joint swelling and preservation of cartilage integrity likely contributed to decreased abnormal activation of nociceptive nerve endings in response to mechanical stimuli.

In the animal experiments, the control group exhibited intact articular cartilage structure with uniform and intense SO staining. In contrast, the OA model group demonstrated markedly reduced SO staining within the cartilage layer and a significantly elevated OARSI score. Following intra‐articular injection of Cur‐MS, these pathological alterations were substantially reversed with a notable restoration of the tissue OARSI score. These results indicate that Cur‐MS exerts a potent protective effect against OA‐associated cartilage degeneration. Furthermore, qPCR analysis revealed that COL2A1 expression was significantly downregulated in the cartilage of the OA group, whereas MMP13 expression was significantly upregulated. Treatment with Cur‐MS not only restored COL2A1 expression but also significantly suppressed MMP13 transcription. Additionally, the study assessed the expression of the macrophage infiltration marker CD68 and key pro‐inflammatory cytokines TNF‐α and IL‐6 in synovial tissue. The expression levels of CD68, TNF‐α, and IL‐6 were significantly reduced in the Cur‐MS group. Collectively, these findings suggest that Cur‐MS can modulate the intra‐articular immune microenvironment by reducing macrophage infiltration and downregulating local pro‐inflammatory cytokine expression.

This study presents a comprehensive transcriptomic analysis of osteoarthritic and normal knee joint cartilage, elucidating transcriptomic characteristics that correspond to the pathological processes of OA including extracellular matrix remodeling, endochondral ossification, and regulation of chondrocyte phenotype. Notably, the core alterations including the upregulation of MMP13 and downregulation of SOX9 are consistently corroborated through differential expression analysis, functional enrichment, single‐gene quantification, and directional comparison, thereby serving as reliable molecular markers of OA‐related cartilage changes. Conversely, the observed directional changes in certain classical inflammatory mediators (IL6, PTGS2, NOS2) and matrix structural genes (COL2A1, ACAN) within this cartilage dataset do not entirely correspond with findings from wet‐laboratory experiments conducted on other tissues or models. This finding does not directly contradict our observations from wet laboratory experiments. Rather, it underscores the complex nature of OA. Firstly, OA comprises a heterogeneous group of diseases characterized by distinct molecular phenotypes, with considerable variability in COL2A1 expression across different subtypes. The data derived from GSE114007 may represent the features of specific subtypes within that cohort. Secondly, chondrocytes demonstrate functional heterogeneity throughout OA progression; certain subpopulations in a compensatory synthetic state may exhibit elevated COL2A1 expression. Single‐cell sequencing analyses have confirmed the existence of multiple chondrocyte subpopulations within OA cartilage, each displaying varying levels of COL2A1 expression. Moreover, alterations at the mRNA level do not necessarily correspond to changes at the protein level, as post‐transcriptional regulatory mechanisms and variations in protein degradation rates can result in discrepancies between mRNA and protein expression. Consequently, differences observed between transcriptomic data and wet laboratory findings should be interpreted within the framework of OA heterogeneity and multi‐level regulatory processes.

The research design employed in this study represents a notable methodological advancement. Traditional OA drug research mainly involves dual validation at the cellular and animal model levels, often neglecting the assessment of drug effects within cartilage microtissues. This study is the first to implement a comprehensive, three‐tiered research model encompassing cells, microtissues and animal models. Such a design not only confirms the extensibility of drug effects but, critically, bridges the translational gap between cellular and animal studies through the use of the pellet model. This innovation enhances the reliability of extrapolating in vitro findings to effective in vivo outcomes. Furthermore, the Cur‐MS utilized herein offers significant technical advantages over free Cur solutions. GelMA, a photocrosslinkable gelatin derivative, integrates the bioactivity of the natural ECM with the tunable properties of synthetic materials. When fabricated into microspheres, GelMA can be administered via minimally invasive injection into the joint cavity. This method accommodates irregular joint defects and provides preliminary structural support for cartilage repair. Additionally, GelMA microspheres serve as drug carriers that substantially prolong Cur retention within the joint cavity, enabling localized and sustained release.

While affirming the findings of this study, it is important to acknowledge its limitations. It is important to note that the 300 μM Cur‐MS concentration employed in this study was selected based on the optimal protective concentration determined through CCK‐8 cell proliferation assays. Nevertheless, the concentration that optimizes cell proliferation and anti‐apoptotic effects in vitro may not correspond to the most effective therapeutic dose for cartilage repair in vivo. Various factors, including drug release kinetics within the microenvironment in vivo, local metabolic processes, and interactions with immune cells, can significantly influence the drug's efficacy. Consequently, the lack of a concentration gradient of Cur in the animal model constitutes a notable limitation of this study. Future investigations should systematically assess the effects of different Cur‐MS concentrations on joint cartilage protection, inflammation suppression, and pain behavior within the same animal model to identify the optimal therapeutic concentration in vivo. A notable limitation of this study is that the degradation behavior of Cur‐MS in vivo was inferred indirectly from in vitro degradation experiments, without direct in situ imaging evidence of degradation. Future investigations should utilize fluorescence labeling or near‐infrared tracing techniques in conjunction with in vivo imaging systems or micro‐CT. These methodologies would enable dynamic, real‐time monitoring of the distribution, retention, and degradation processes of microspheres within the joint cavity, thereby facilitating a more precise correlation between in vitro release profiles and in vivo degradation. Besides, the in vitro experiments conducted in this study predominantly utilized animal chondrocytes rather than primary human chondrocytes. Given the limited availability and inherent variability of human chondrocytes, and considering that the primary objective of this research was to assess the feasibility of the Cur‐MS formulation and its progressive effects across multiple models, validation using primary human chondrocytes represents a critical direction for future investigation. Subsequent studies should employ primary human chondrocytes obtained from OA patients or healthy donors to evaluate the protective effects of Cur‐MS at an individual level, thereby strengthening its clinical translational potential. Additionally, the development of responsive microsphere systems capable of programmed or on‐demand release represents a promising avenue for enhancing OA treatment. Although this research preliminarily confirmed the local safety of Cur‐MS both in vitro and in animal models, as a preclinical investigation, more comprehensive pharmacokinetic analyses and long‐term in vivo toxicity assessments remain necessary. Furthermore, there is potential for optimization regarding administration methods and formulation design. For instance, targeted modification of microspheres and the incorporation of combination therapies with other drugs represent promising avenues to further enhance the overall efficacy of synergistic treatment.

## 5 Conclusion

In summary, this study successfully developed an injectable GelMA hydrogel microsphere delivery system encapsulating Cur. Concurrently, an innovative multi‐tiered systematic validation framework was established, incorporating chondrocytes, cartilage microtissue and animal models. This comprehensive evaluation model elucidated the multi‐level cartilage‐protective effects and immunomodulatory mechanisms of Cur‐MS, spanning from the cellular and tissue levels to in vivo conditions. Cur‐MS synergistically inhibit NF‐κB‐mediated inflammatory activation, mitigate oxidative stress induced damage, suppress pro‐hypertrophic signaling pathways, enhance cartilage‐specific ECM synthesis, and remodel the intra‐articular immune microenvironment. Across cellular, pellet and animal model systems, pathological alterations were restored to near‐normal states. Given its notable efficacy, favorable biocompatibility, and the advantages of minimally invasive injectable delivery, Cur‐MS represents a promising drug delivery strategy, offering novel insights and experimental evidence for disease‐modifying OA therapies. Future investigations will further delineate the molecular target network of Cur and rigorously evaluate the translational potential of this delivery system.

## Author Contributions

**Xin Jiang:** data curation, investigation, writing – original draft, software. **Bin Jiang:** writing – original draft, conceptualization, methodology, investigation, formal analysis, data curation. **Hao Niu:** writing – review and editing, visualization, resources. **Shaobo Li:** writing – original draft, supervision, methodology, software. **Wenbin Ji:** conceptualization, writing – review and editing, project administration, resources, validation, formal analysis.

## Funding

The authors have nothing to report.

## Ethics Statement

The experimental protocol was approved by Qilu Hospital of Shandong University (Qingdao) Experimental Animal Ethics Committee and Qingdao Harwars Biotechnology Co. Ltd. Experimental Animal Ethics Committee. The approval number is AUP‐20250823‐001.

## Conflicts of Interest

The authors declare no conflicts of interest.

## Supporting information


**Figure S1:** Cell viability assay. (A) Chondrocytes of free Cur with different concentration were evaluated for cell viability using live/dead staining. (B) Quantitative analysis of the percentage of viable cells. Scale bar = 200 μm; ns indicates no statistical significance; ***p < 0.001; ****p < 0.0001.

## Data Availability

The data that support the findings of this study are available from the corresponding authors upon reasonable request.
