# Supplementary material for: Curcumin‐Loaded GelMA Microspheres Alleviate Osteoarthritis: Transcriptomic Evidence for Immune Microenvironment Remodeling and ECM Homeostasis Restoration
Source: Chem Biol Drug Des. 2026 Aug 2;108(2):e70373. doi: 10.1111/cbdd.70373 (PMC13430147; doi:10.1111/cbdd.70373)
Supplement: Supplementary file 1 — Figure S1: Cell viability assay. (A) Chondrocytes of free Cur with different concentration were evaluated for cell viability using live/dead staining. (B) Quantitative analysis of the percentage of viable cells. Scale bar = 200 μm; ns indicates no statistical significance; ***p < 0.001; ****p < 0.0001. [file CBDD-108-e70373-s001.docx]

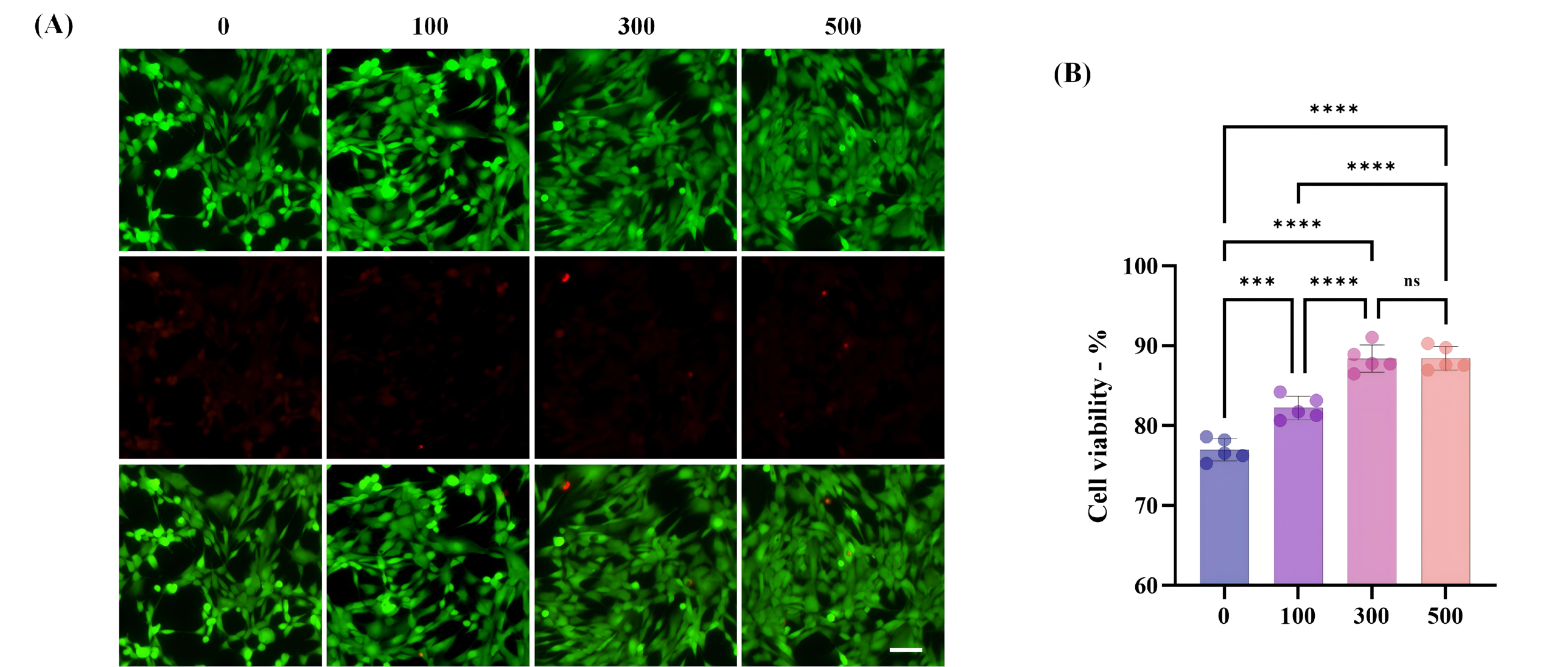


Figure S1. Cell viability assay. (A) Chondrocytes of free Cur with different concentration were evaluated for cell viability using live/dead staining. (B) Quantitative analysis of the percentage of viable cells. Scale bar = 200 μm; ns indicates no statistical significance; ***P < 0.001; ****P < 0.0001.
